# Mapping Reef Fish and the Seascape: Using Acoustics and Spatial Modeling to Guide Coastal Management

**DOI:** 10.1371/journal.pone.0085555

**Published:** 2014-01-15

**Authors:** Bryan Costa, J. Christopher Taylor, Laura Kracker, Tim Battista, Simon Pittman

**Affiliations:** 1 Biogeography Branch, National Centers for Coastal Ocean Science, National Oceanic and Atmospheric Administration, Silver Spring, Maryland, United States of America; 2 Consolidated Safety Services, Fairfax, Virginia, United States of America; 3 Center for Coastal Fisheries and Habitat Research, National Centers for Coastal Ocean Science, National Oceanic and Atmospheric Administration, Beaufort, North Carolina, United States of America; 4 Marine Institute, University of Plymouth, Plymouth, United Kingdom; University of Waikato (National Institute of Water and Atmospheric Research), New Zealand

## Abstract

Reef fish distributions are patchy in time and space with some coral reef habitats supporting higher densities (i.e., aggregations) of fish than others. Identifying and quantifying fish aggregations (particularly during spawning events) are often top priorities for coastal managers. However, the rapid mapping of these aggregations using conventional survey methods (e.g., non-technical SCUBA diving and remotely operated cameras) are limited by depth, visibility and time. Acoustic sensors (i.e., splitbeam and multibeam echosounders) are not constrained by these same limitations, and were used to concurrently map and quantify the location, density and size of reef fish along with seafloor structure in two, separate locations in the U.S. Virgin Islands. Reef fish aggregations were documented along the shelf edge, an ecologically important ecotone in the region. Fish were grouped into three classes according to body size, and relationships with the benthic seascape were modeled in one area using Boosted Regression Trees. These models were validated in a second area to test their predictive performance in locations where fish have not been mapped. Models predicting the density of large fish (≥29 cm) performed well (i.e., AUC = 0.77). Water depth and standard deviation of depth were the most influential predictors at two spatial scales (100 and 300 m). Models of small (≤11 cm) and medium (12–28 cm) fish performed poorly (i.e., AUC = 0.49 to 0.68) due to the high prevalence (45–79%) of smaller fish in both locations, and the unequal prevalence of smaller fish in the training and validation areas. Integrating acoustic sensors with spatial modeling offers a new and reliable approach to rapidly identify fish aggregations and to predict the density large fish in un-surveyed locations. This integrative approach will help coastal managers to prioritize sites, and focus their limited resources on areas that may be of higher conservation value.

## Introduction

The rapid emergence of place-based management strategies, such as marine protected areas (MPAs), has increased the demand for reliable information describing the distribution of fish across large portions (i.e., 10 s to 100 s km^2^) of the ocean [1], [2], [3]. Patchiness of fish populations in time and space, combined with resource constraints on management, often requires that coastal managers identify spatial priorities. A common strategy to identify these priorities is to select locations of high conservation value based on biological characteristics and relative vulnerability [2], [4], [5], [6]. Fish aggregations (i.e., locations where a suite of environmental conditions interact to support high densities of fish) are typically given high priority in MPA network design and marine spatial planning [7], [8], [9]. However, locating and characterizing fish aggregations can be challenging over broad geographic areas (i.e., 10 s to 100 s km^2^), especially when they occur in waters too deep for surveys using conventional SCUBA diving; where turbidity impairs visual surveys; or when aggregations are transient and only detectable at night. Additional challenges arise when animal distribution patterns need to be expanded from fine-scale visual surveys (covering <100 m^2^) to broader spatial scales (covering 10 s to 100 s km^2^) that are operationally relevant to coastal and marine management [10], [11], [12]. Scaling up patterns from fine-scale surveys is challenging because there is no single scale at which ecological patterns should be studied since organisms show variability at a range of spatial, temporal, and organizational scales [13].

Although underwater acoustic technology is not new, rarely have coral reef ecosystem studies simultaneously mapped and quantified the locations and size of fish along with the three-dimensional structure of the surrounding seafloor. These data provide an opportunity to model fish-seascape relationships at multiple spatial scales that are appropriate for studies of highly mobile organisms. In both tropical and temperate waters, seafloor structure (derived from bathymetry) has been established as a useful predictor of fish distributions [9], [14], [15]. Water depth and seafloor topography (e.g., rugosity, slope-of-the-slope, slope, curvature), sometimes combined with relative across-shelf position, have repeatedly emerged as excellent predictors for fish [9], [14], [15], [16]. These data can be analyzed to produce descriptive maps of fish distributions for discrete size-classes, providing a unique opportunity to bridge the informational gap between ecology and management [17].

**Figure 1:**
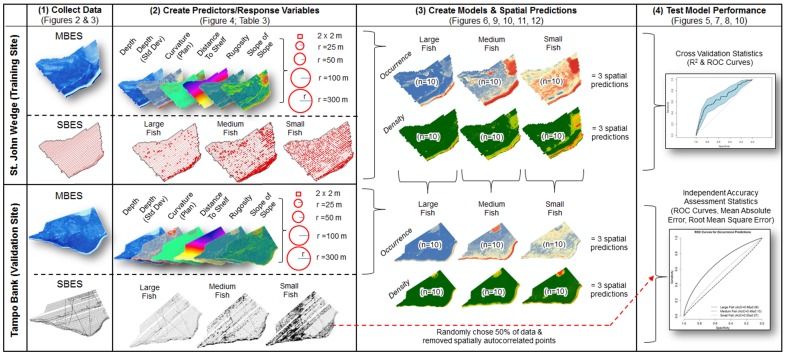
Diagram of modeling process. Steps used to train and validate the Boosted Regression Tree models developed from the SBES and MBES datasets.

The importance of structural complexity for maintaining the integrity and function of coral reef ecosystems is well established at fine spatial scales (i.e., centimeters to meters) [18], [19], [20]. The importance of this complexity at broader spatial scales is less well studied and understood. To address this research gap, we quantified seafloor structure (and distance to the shelf edge) at multiple spatial scales to explore how fish in different sizes classes are distributed across the seascape [20], [21], [22]. We acquired spatially and temporally coincident data with a multibeam echosounder (MBES) to map seafloor terrain and a splitbeam echosounder (SBES) to map fish in the water column in two areas in the U.S. Virgin Islands (USVI). These two areas were chosen because they contained spawning aggregation sites for commercially important snapper and grouper species. We then used Boosted Regression Trees (BRT), a machine learning algorithm, and Geographic Information Systems (GIS) software, to model and map the spatial relationship between seafloor structure and the density of fishes in three size classes (Fig. 1). The key questions addressed were:

1. Which seafloor structure and distance surfaces were the best predictors of fish occurrence and density?
2. Does fish-habitat data derived from acoustic sensors provide sufficient information to develop useful spatial predictions of fish distributions across the seascape?

## Methods

### Study Areas

**Figure 2:**
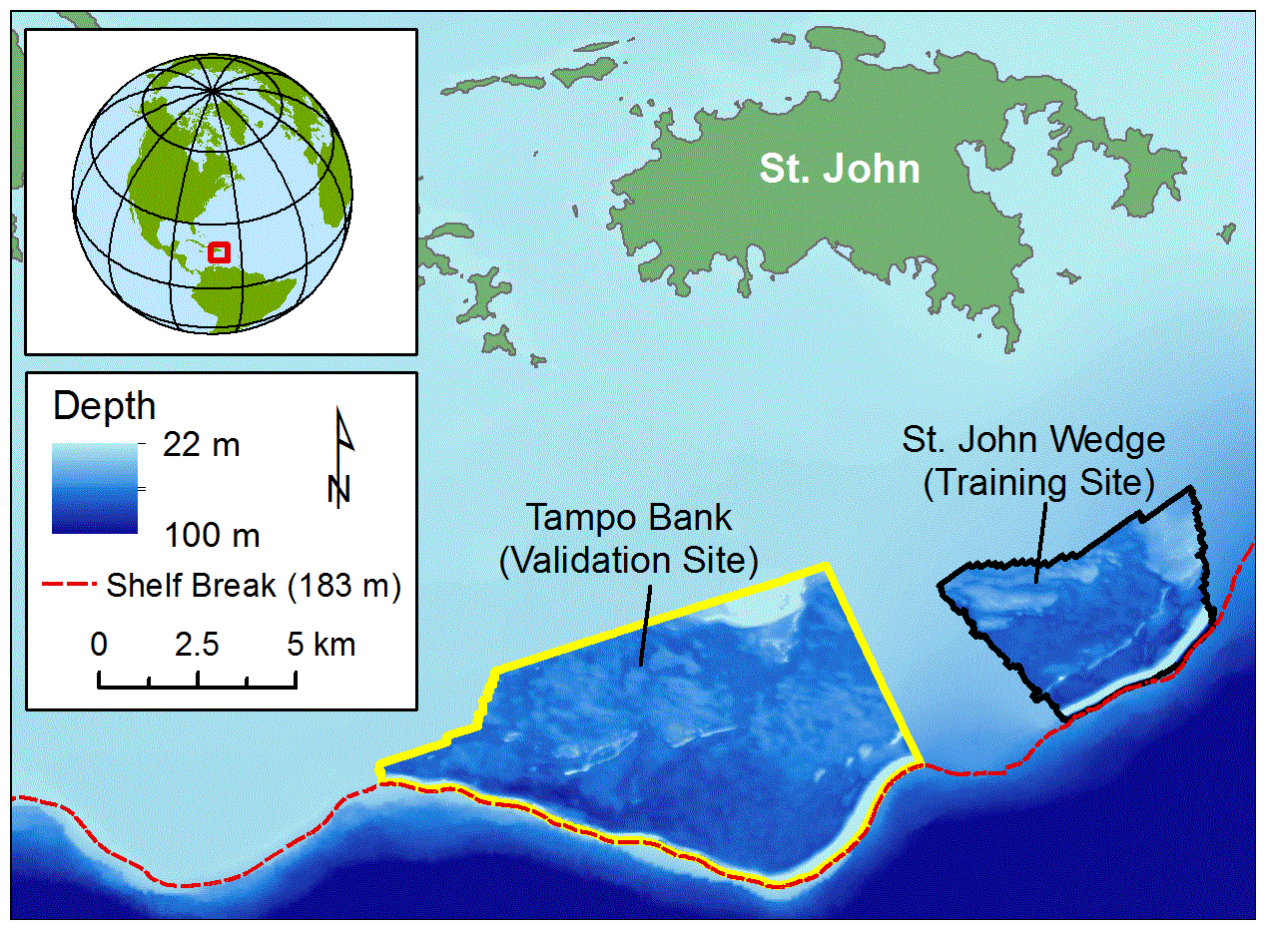
Map of study areas. Location of the study sites in the U.S. Virgin Islands. The spatial predictions were developed in one site (St. John Wedge) and validated in the other site (Tampo Bank).

This research was conducted in two areas (i.e., St. John Wedge and Tampo Bank) south of St. John in the USVI (Fig. 2). St. John Wedge is 22 km^2^ and Tampo Bank is 62 km^2^. Both areas were mapped using multibeam and splitbeam echosounders. No specific permissions were required to survey these locations because they are not actively managed by the territorial or federal government, and this study did not involve endangered or protected species. These areas were chosen because they were in close proximity to known spawning sites (i.e., Grammanik Bank) for commercially important fish species (e.g., *Ocyurus chrysurus* and *Epinephelus guttatus*) [9], [6]. Tampo Bank is also suspected to be spawning site for Mutton Snapper (*Lutjanus analis*) [23]. BRT models were trained in St. John Wedge, and validated in Tampo Bank. Depths in these two areas ranged from 22 to 100 m (Table 1), although over 70% of each site was shallower than 55 m. Fish density measurements ranged from 0 to 33 fish per 100 m^2^.

**Table 1:** Depths in the training and validation sites.

Depths	% Area	
(m)	St. John Wedge	Tampo Bank
22<35	3.7	1.8
35<45	16.7	3.8
45<55	73.0	64.8
55<65	5.7	29.2
65<75	0.4	0.4
75<85	0.3	0.0
85<100	0.3	0.0

Depths found in St. John Wedge and Tampo Bank ranged from 22 to 100 m. However, greater than 70% of both areas were less than 55 meters deep.

### Mapping Fish and the Seafloor

#### Mapping fish using a splitbeam echosounder

SBES data describing fish sizes, densities and distributions were collected in St. John Wedge from March 29 – April 16, 2011, and in Tampo Bank from March 18 – April 6, 2010 using a Simrad EK-60 (120 kHz) splitbeam echosounder. This scientific echosounder was calibrated using a tungsten carbide sphere, allowing for accurate measurements of fish size. Splitbeam echosounders detect fish in the water column by rapidly transmitting pulses of sound (pings) that reflect off objects of differing densities than the surrounding water. An internal air filled sac (called the swim bladder) is the primary contributor to a fish's acoustic reflection [24]. Larger fish reflect more sound because they have larger swim bladders. Survey lines were acquired parallel to depth contours, and spaced to provide complete MBES coverage of the seafloor. Only a small percentage of each survey line was sampled by the SBES because it had a narrower swath (about 7°) than the MBES (about 120°).

SBES data were processed to detect individual fish using Echoview software version 5.3. The water-seafloor interface was delineated to separate this acoustic signal from fish detections. Other acoustic interference (e.g., air bubbles) and faint echoes, likely representing plankton and other non-fish targets, were masked or eliminated from the data. Vessel speed (ca 7 kts) and rapid ping rate (3–8 pulses s^−1^) resulted in multiple, sequential echoes from each individual fish. These sequential echoes were grouped using a target tracking algorithm [25] and retained as individual fish targets. Each fish was assigned a central geographic position, a depth below the water surface and average, calibrated target strength measured in decibels (dB). Very few controlled observations are available to determine specific relationships between target strength and total length for coral reef fish [26]. Here, we used a species-independent, generalized formula to convert target strength into fish length [9], [27]. Data along the survey path were binned into 100 m^2^ intervals to normalize for the variation in beam width caused by changing depths. Fish densities were calculated for all fish exceeding −50 dB or a length of about 6 cm. The final dataset was exported as an ArcGIS point shapefile (referenced to North American Datum 1983 Universal Transverse Mercator 20 North) with each point representing the centroid of a 100 m^2^ bin.

**Figure 3:**
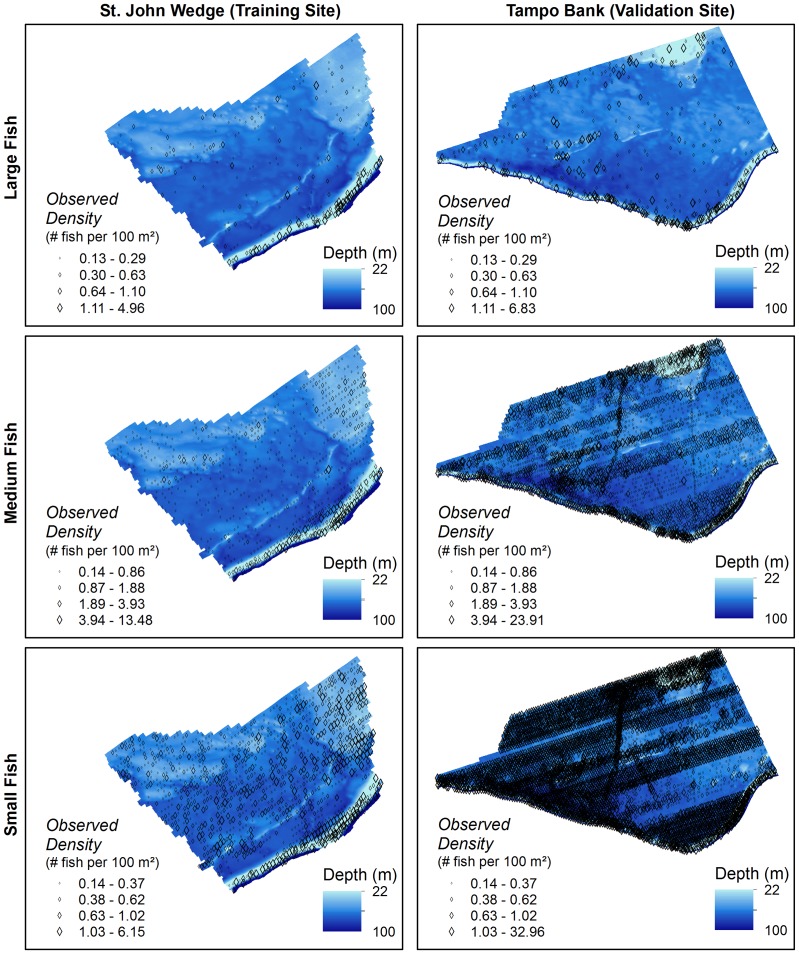
Maps of SBES data. Presence and density of large, medium and small fish in the training site (left) and validation site (right).

The species of individual fish cannot be identified from a single SBES frequency. Instead, fish targets were sorted into three size classes with the goal of separating them into ecological groups. These groups were initially chosen based on size estimates for species and groups from visual censuses for fish communities in the region [9], [28]. Large fish (≥29 cm) are comprised of many important fishery species (e.g., *Serranidae* and *Lutjanidae*) and other large predatory species (Table 2) [28]. Medium fish (12–28 cm) include large reef residents and juvenile or small adults of fishery species (Table 3) [28]. Small fish (≤11 cm) represent small reef resident species, small planktivores and possibly juveniles of fishery species (Table 4) [28]. Maps of fish density were created for each size class in the training and validation sites (Fig. 3).

**Table 2:** Large fish (≥29 cm) commonly found <55 m deep around St. John.

Number	Species Scientific Name	Species Common Name	Inhabited Depths (m)	Preferred Habitat
1	*Caranx crysos*	Blue runner	<100	water-column/seafloor (hardbottom)
2	*Lutjanus griseus*	Gray snapper	0–180	seafloor (hardbottom)
3	*Epinephelus guttatus*	Red hind	2–100	seafloor (hardbottom)
4	*Ocyurus chrysurus*	Yellowtail snapper	10–180	water-column/seafloor (hardbottom)
5	*Lutjanus analis*	Mutton snapper	40–95	water-column/seafloor (hardbottom)
6	*Lutjanus apodus*	Schoolmaster	2–63	water-column/seafloor (hardbottom)
7	*Pomacanthus paru*	French angelfish	3–100	water-column/seafloor (hardbottom)
8	*Cephalopholis fulva*	Coney	2–150	seafloor (hardbottom)
9	*Melichthys niger*	Black durgeon	0–75	water-column/seafloor (hardbottom)
10	*Bodianus rufus*	Spanish hogfish	3–70	seafloor (hardbottom)

Species of large fish commonly found in depths <55 m around St. John. The 55 m cutoff was used because >70% of both the training and validation sites were shallower than this depth. The most commonly observed species were identified from surveys conducted from 2001 to 2011 around St. John [28]. These fish species may represent the species of fish detected in the SBES data.

**Table 3:** Medium-sized fish (12–28 cm) commonly found <55 m deep around St. John.

Number	Species Scientific Name	Species Common Name	Inhabited Depths (m)	Preferred Habitat
1	*Clepticus parrae*	Creole wrasse	8–100	water-column/seafloor (hardbottom)
2	*Haemulon flavolineatum*	French grunt	0–60	seafloor (hardbottom)
3	*Cephalopholis fulva*	Coney	2–150	seafloor (hardbottom)
4	*Halichoeres garnoti*	Yellowhead wrasse	4–60	seafloor (hardbottom)
5	*Ocyurus chrysurus*	Yellowtail snapper	10–70	water-column/seafloor (hardbottom)
6	*Decapterus macarellus*	Mackerel scad	40–200	water-column
7	*Pseudupeneus maculatus*	Spotted goatfish	0–90	seafloor (softbottom)
8	*Epinephelus guttatus*	Red hind	2–100	seafloor (hardbottom)
9	*Lutjanus apodus*	Schoolmaster	2–63	water-column/seafloor (hardbottom)
10	*Myripristis jacobus*	Blackbar soldierfish	2–100	water-column/seafloor (hardbottom)

Species of medium-sized fish commonly found in depths <55 m around St. John. The 55 m cutoff was used because >70% of both the training and validation sites were shallower than this depth. The most commonly observed species were identified from surveys conducted from 2001 to 2011 around St. John [28]. These fish species may represent the species of fish detected in the SBES data.

**Table 4:** Small fish (≤11 cm) commonly found <55 m deep around St. John.

Number	Species Scientific Name	Species Common Name	Inhabited Depths (m)	Preferred Habitat
1	*Stegastes partitus*	Bicolor damselfish	0–100	seafloor (hardbottom)
2	*Chromis cyanea*	Blue chromis	10–60	water column/seafloor (hardbottom)
3	*Halichoeres garnoti*	Yellowhead wrasse	4–80	seafloor (hardbottom)
4	*Serranus tortugarum*	Chalk bass	8–90	water column/seafloor (hardbottom)
5	*Clepticus parrae*	Creole wrasse	8–100	water column/seafloor (hardbottom)
6	*Chromis multilineata*	Brown chromis	0–60	water column/seafloor (hardbottom)
7	*Sparisoma atomarium*	Greenblotch parrotfish	20–55	seafloor (hardbottom/softbottom)
8	*Cryptotomus roseus*	Bluelip parrotfish	0–60	seafloor (softbottom)
9	*Ocyurus chrysurus*	Yellowtail snapper	10–180	water column/seafloor (hardbottom)
10	*Gramma loreto*	Fairy basslet	1–60	seafloor (hardbottom)

Species of small fish commonly found in depths <55 m around St. John. The 55 m cutoff was used because >70% of both the training and validation sites were shallower than this depth. The most commonly observed species were identified from surveys conducted from 2001 to 2011 around St. John [28]. These fish species may represent the species of fish detected in the SBES data.

#### Mapping the seafloor using a multibeam echosounder

Bathymetry (i.e., depth) was collected concurrently with the SBES data in St. John Wedge and Tampo Bank using a hull-mounted Reson SeaBat 7125 SV1 MBES system [29], [30]. MBES systems measure seafloor depth by transmitting multiple pulses of sound several times a second and then recording the time and angle of the acoustic returns. These two pieces of information are used to create highly resolved images of seafloor depth and topography. Each study area was mapped using the 400 kHz frequency, producing 2×2 m depth surface. Depth surfaces were corrected for sensor offsets, latency, roll, pitch, yaw, static draft, influence of tides and the changing speed of sound in the water column. Both surfaces met International Hydrographic Organization Order 1 standards [31], and had a maximum horizontal uncertainty of ±10.0 m and vertical uncertainty of ±1.39 m. All data were referenced to North American Datum 1983 Universal Transverse Mercator 20 North projection and Mean Lower Low Water vertical coordinate system.

**Figure 4:**
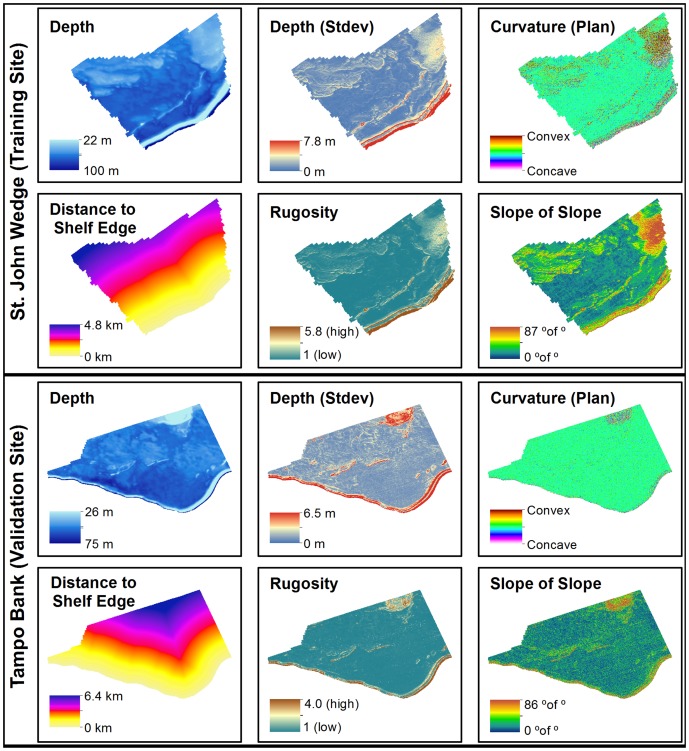
Maps of predictor data. Six environmental variables used as predictors in both the training and validation sites. These six variables were also included in the modeling process at four additional spatial scales. A total of 24 predictors were used to create each boosted regression tree model and spatial prediction.

Surfaces describing the three dimensional structure of the seafloor were derived from these depth surfaces using ArcGIS's Spatial Analyst Toolbox and DEM Surface Toolbox [32] (Fig. 4). The surfaces, including standard deviation of depth, plan (or cross-sectional) curvature, rugosity and slope of slope (Table 5), were selected based on their demonstrated utility for predicting coral reef fish abundances and distributions [14], [18]. The Spatial Analyst Toolbox was also used to calculate the geographic distance of the center of each grid cell to the shelf edge (i.e., the 183 m isobath). These six surfaces (i.e., depth, standard deviation of depth, plan curvature, rugosity, slope of slope, and distance to the shelf edge) were computed at four additional spatial scales (i.e., mean values within a radius of 25, 50, 100 and 300 m) to examine the influence of scale on fish distributions. These spatial scales were chosen based on previous research, which showed strong fish-seascape relationships at similar spatial scales [14], [18], [33], [34]. A total of 24 predictors (i.e., 6 surfaces x 4 spatial scales) were included in the modeling process and used to develop spatial predictions in the training and validation sites.

**Table 5:** Descriptions of predictors.

Predictor Dataset	Unit	Description	Tool Used
1. Depth	Meters	Water depth	-
2. Depth (Standard Deviation)	Meters	Dispersion of water depth values about the mean (in a 3×3 cell neighborhood)	Focal statistic function in ArcGIS's Spatial Analyst
3. Curvature (Plan or Cross-Sectional)	Concave (−) & Convex (+)	Curvature of the surface perpendicular to the slope direction (in a 3×3 cell neighborhood)	Curvature function in ArcGIS's Spatial Analyst
4. Distance to Shelf Edge	Kilometers	Distance of the centroid of each pixel to the 183 m (100 fathom) isobath	Euclidean distance function in ArcGIS's Spatial Analyst
5. Rugosity	Ratio value	Ratio of surface area to planar area (in a 3×3 cell neighborhood)	Surface Area and Ratio function in DEM Surface Toolbox
6. Slope of the Slope	Degrees of degrees	Maximum rate of maximum slope change (in a 3×3 cell neighborhood)	Slope function in ArcGIS's Spatial Analyst

Environmental variables used to predict large, medium and small fish occurrence and density. Each variable was also included in the modeling process at four additional spatial scales (i.e., using circles with radii of 25, 50, 100 and 300 m).

### Predicting Fish Distributions and Densities

#### Boosted regression trees

Boosted regression trees (BRT) is a machine learning technique used effectively in ecology to model the complex, non-linear relationships between organisms and their environment. BRTs model these complex relationships by developing many (sometimes hundreds to thousands) simple models based on random subsets of the data [35], [36]. These simple models are then combined linearly to produce one final aggregate (i.e., ensemble) model [37]. The fitted values in this ensemble model are more stable than values from an individual model, improving its overall predictive performance [37], [38]. The BRT approach to spatial modeling was used in this study because it can deal with data that is not normally distributed, is robust to missing data values, can handle interactions among predictors and compared favorably (both in terms of predictive performance and accuracy) to other modeling techniques [14], [37], [38].

#### Model development

For this study, 60 BRT models were generated from MBES and SBES data. Ten of these models predicted large fish occurrence, 10 predicted medium fish occurrence, 10 predicted small fish occurrence, 10 predicted large fish density, 10 predicted medium fish density, and 10 predicted small fish density. Multiple models for occurrence and density were created to avoid fitting one model too closely to the data, and to better understand and quantify the stability of BRT's variable selection and predictive performance [37]. BRT models were developed using the “gbm.step” function in the “dismo” package version 0.7 [39] implemented in R software version 2.15. Each BRT model was trained using a different random 50% of the St. John Wedge SBES data (n = 1,641 points representing 100 m^2^ bins). The remaining 50% were used for cross validation (CV).

In each of the St. John Wedge and Tampo Bank areas, six predictive surfaces (i.e., three surfaces predicting fish occurrence by size class and three predicting fish density by size class) were produced by averaging each group of 10 BRT models. Spatial predictions were developed using the “raster” package version 2.0 [40] implemented in R version 2.15. Spatial predictions for fish occurrence denote the probability that a large, medium or small fish is present in a 2×2 m area. Spatial predictions for fish density denote the number of large, medium and small fish predicted to be in a 2×2 m area. These spatial predictions were independently validated using the Tampo Bank SBES data to simulate and evaluate how well they would perform in areas that had not been surveyed with a SBES.

#### Model performance

When evaluating a model's performance, both its discrimination capacity and the reliability should be assessed. Discrimination capacity refers to the ability of the model to differentiate between classes (e.g., presences and absences), while reliability describes the agreement between the predicted and observed values at specific locations [41], [42]. The discrimination capacity of the BRT models for large, medium and small fish occurrence and density was assessed using receiver operating characteristic (ROC) curves, and their reliability was evaluated using mean absolute error (MAE) and root mean square error (RMSE). MAE and RMSE both measure the average magnitude of the predictive errors (independent of their direction). However, MAE weights each error equally in the average, while RMSE weights large errors much more heavily. Both metrics are reported here so that the impact on different management applications can be explored.

The other model performance metric, called ROC curves, measure a model's performance by comparing its sensitivity (i.e., true positive prediction rate) to its specificity (i.e., false positive prediction rate) over the continuous range of predicted values. The diagonal y = x line in a ROC curves denotes how a randomly generated model would perform. ROC curves above this line perform better than a random model, while ROC curves below this line have useful information but are applying it incorrectly [43]. The area under the curve (AUC) statistic was also calculated, which describes the overall predictive performance of a model compared to a random guess. It is equal to the probability that a model will rank a randomly chosen presence higher than a randomly chosen absence. AUC values ranging from 0.5 to 0.6 suggest the model is no better at discriminating classes than random chance; values from 0.6 to 0.7 denote “poor” model performance; values ranging from 0.7 to 0.8 denote “acceptable” model performance; values from 0.8 to 0.9 denote “excellent” model performance, and values greater than 0.9 denote “outstanding” model performance [44].

ROC curves have several advantages over traditional accuracy assessment techniques, including confusion matrices. One notable advantage is that ROC curves are independent of binary thresholds (i.e., break points where animals are defined as present or absent) that are often chosen subjectively [45], [46]. ROC curves do not require that a predictive threshold be selected because they describe a model's performance over the complete range of predicted values. The other important advantage of ROC curves is that they are unaffected by changes in animal prevalence (i.e., unequal amounts of presences and absences) [45], [46] because they are based on ratios (and not summaries) of true presences to false presences. This independence is particularly important when developing models for rare animals (i.e., that have low prevalence, like large fish) because it is possible to get high overall model accuracy by predicting such animals are absent everywhere [45].

#### Evaluating model performance

ROC curves and correlation coefficients were developed in R using 10-fold cross validation data in the St. John Wedge area. In the Tampo Bank area, ROC curves were developed (along with MAE and RMSE) using an independent SBES dataset. This independent dataset (n = 5,269) was used solely for assessing the performance of the final spatial predictions. A subset of this validation dataset (n = 2,634) was chosen randomly to avoid biasing the evaluation process in R. Spatially autocorrelated points were then removed from this data subset because positive autocorrelation violates the assumption of independence and biases statistical tests by effectively overestimating the true sample size [47], [48].

These autocorrelated points were identified in the large, medium and small fish datasets by detrending them using local polynomial regression, developing three empirical semi-variograms from the residuals, and fitting spherical models to the variograms using the “stats” and “geoR” package version 1.7 in R [49], [50]. The ranges for the large, medium and small fish variogram models were 280 m, 503 m and 272 m, respectively. Points closer together than these distances were assumed to be spatially autocorrelated, and were removed from the validation process using Matlab. This step removed 2201, 2479 and 2219 points from the large, medium and small fish validation datasets. The remaining 432, 154 and 415 spatially independent points were used to create ROC curves and calculate MAE and RMSE for the large, medium and small fish occurrence and density predictions.

ROC curves for the fish density predictions were created differently than those for the fish occurrence predictions because ROC curves are not designed to handle validation data that is continuous (i.e., densities). To address this issue, the large, medium and small fish density data was divided into four classes (i.e., absent to low, low, medium and high) using Jenks natural breaks in ArcGIS (Tables 6, 7, 8). This method was chosen because it is well suited for grouping data with large variances [51]. Six ROC curves were then created for each of the large, medium and small fish density predictions by comparing the four density classes in a pair-wise fashion (i.e., absent vs. low, absent vs. medium, absent vs. high, low vs. medium, low vs. high and medium vs. high). AUC was calculated for each curve as well as for the entire multiclass prediction using the method defined by Hand and Till, 2001 [52].

**Table 6:** Frequency of Large Fish Records by Density Class.

Fish Density Class	Fish Density Threshold (# fish/100 m^2^)	Percent of Total Records (Training)	Percent of Total Records (Validation)
Absent to Low	≤0.29	93.1%	91.9%
Low	0.30 – 0.63	4.0%	5.0%
Medium	0.64 –1.10	1.2%	1.6%
High	≥1.11	1.8%	1.5%

Frequency of large fish records by density class in both the training and validation areas. These classes were determined using Jenks Natural Breaks.

**Table 7:** Frequency of Medium Fish Records by Density Class.

Fish Density Class	Fish Density Threshold (# fish/100 m^2^)	Percent of Total Records (Training)	Percent of Total Records (Validation)
Absent to Low	≤0.86	89.5%	64.0%
Low	0.87 – 1.88	6.3%	18.8%
Medium	1.89 – 3.93	2.9%	12.4%
High	≥3.94	1.3%	4.8%

Frequency medium fish records by density class in both the training and validation areas. These classes were determined using Jenks Natural Breaks.

**Table 8:** Frequency of Small Fish Records by Density Class.

Fish Density Class	Fish Density Threshold (# fish/100 m^2^)	Percent of Total Records (Training)	Percent of Total Records (Validation)
Absent to Low	≤0.37	62.7%	35.4%
Low	0.38 – 0.62	18.0%	5.6%
Medium	0.63 – 1.02	10.8%	7.1%
High	≥1.03	8.5%	51.9%

Frequency of small fish records by density class in both the training and validation areas. These classes were determined using Jenks Natural Breaks.

#### Spatial distribution of model errors

While ROC curves, MAE and RMSE describe the discrimination capacity and reliability of models, they do not describe the spatial distribution of model errors [53], [54]. Analyzing the spatial location and arrangement of errors can be important because they may offer clues about missing ecological or biological variables and their spatial structure [54]. A model with spatially clustered errors (versus randomly distributed) may indicate that there are unaccounted for spatially structured variables. To better understand this spatial structure, the large, medium and small fish validation datasets were subtracted from their corresponding occurrence and density predictions. Cluster and outlier analysis in ArcGIS's Spatial Analyst Toolbox was then used to describe the spatial distribution and clustering of the residual model errors. This tool identifies statistically significant spatial clusters of high values, low values and outliers using inverse distance weighting and the Anselin Local Moran's I statistic.

#### Contribution of predictor variables

Two different metrics were used to quantify how much each predictor contributed to the BRT models. The first metric (i.e., ‘the relative influence of each predictor variable’) is based on the number of times that a predictor is selected for splitting. This sum is weighted by how much the model is improved by each split, averaged across all the trees and scaled so that the sum equals 100 [37]. The higher the scaled number, the more influence a predictor has on the model and vice versa. The top three predictors from this analysis were examined in the discussion for each spatial prediction. The second metric (i.e., ‘partial dependence plots’) examines how fish occurrences and densities change over the continuous range of values for a predictor (after accounting for the average effects of all other predictors in the model). These plots can be used to identify thresholds or peaks in the presence and density of large, medium and small fish for each predictor [37].

## Results

### Fish Occurrence Models

#### Large fish

**Figure 5:**
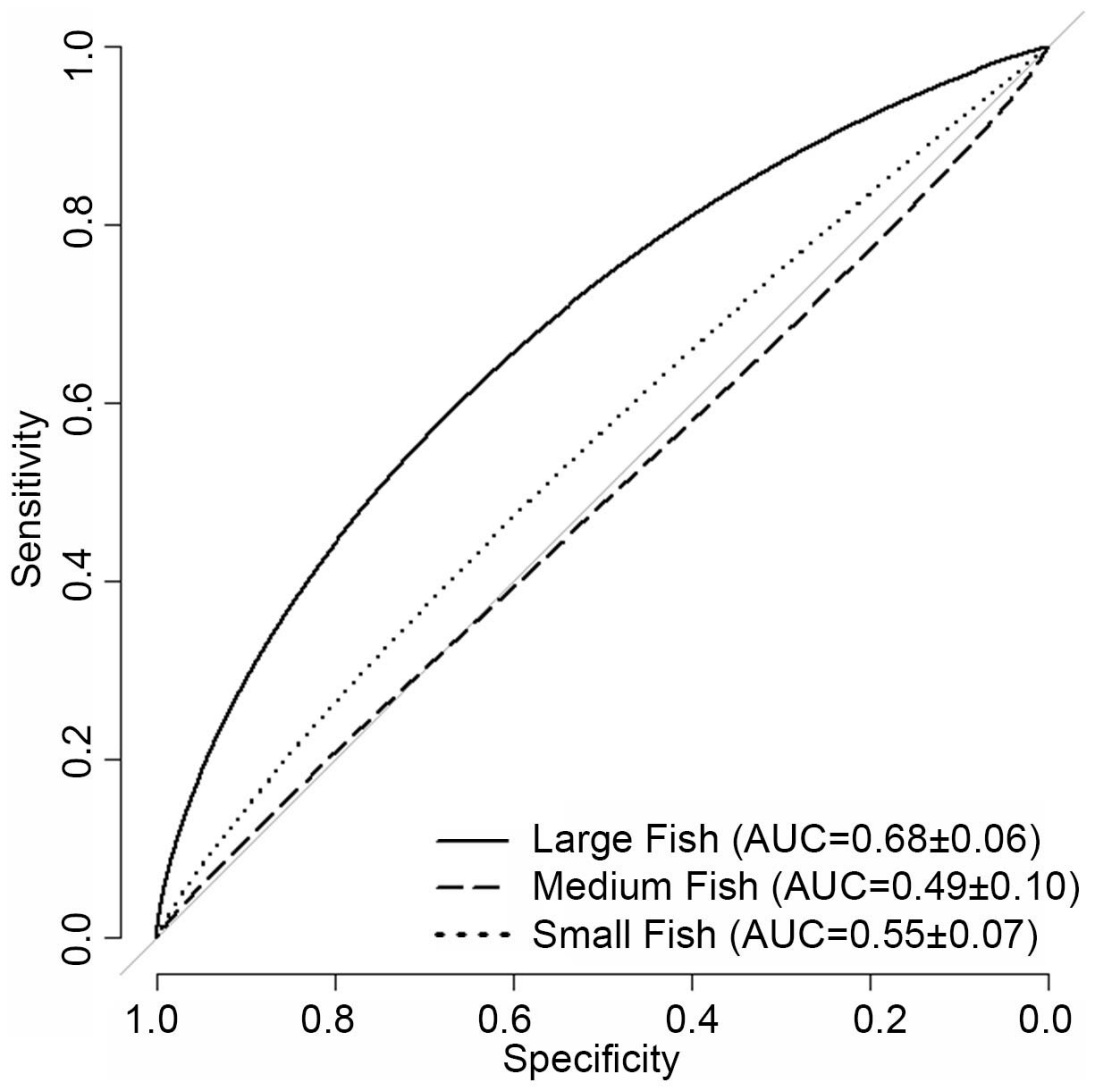
ROC curve for fish occurrence predictions. These ROC curves were developed using the independent dataset in the Tampo Bank area. Area under the curve (AUC) values (at the 95% confidence interval) are listed in the lower right hand corner.

**Figure 6:**
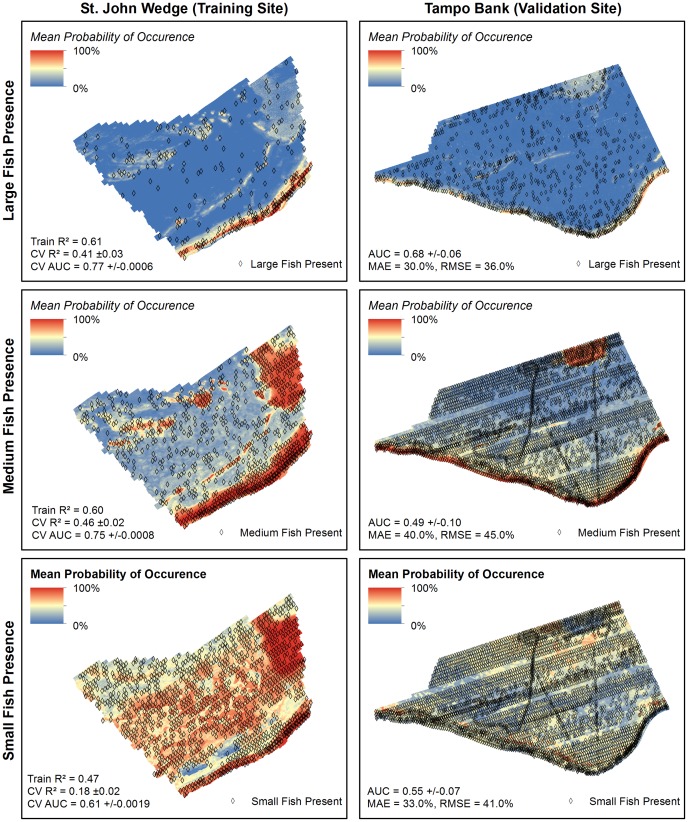
Fish occurrence spatial predictions. Three spatial predictions denoting the probability of occurrence for large, medium and small fish were created for both the training and validation sites. The spatial outputs from 10 different BRT models were averaged to create the spatial predictions seen here. Metrics describing the performance and accuracy of these models and spatial predictions are located in the lower left corner of each map.

**Figure 7:**
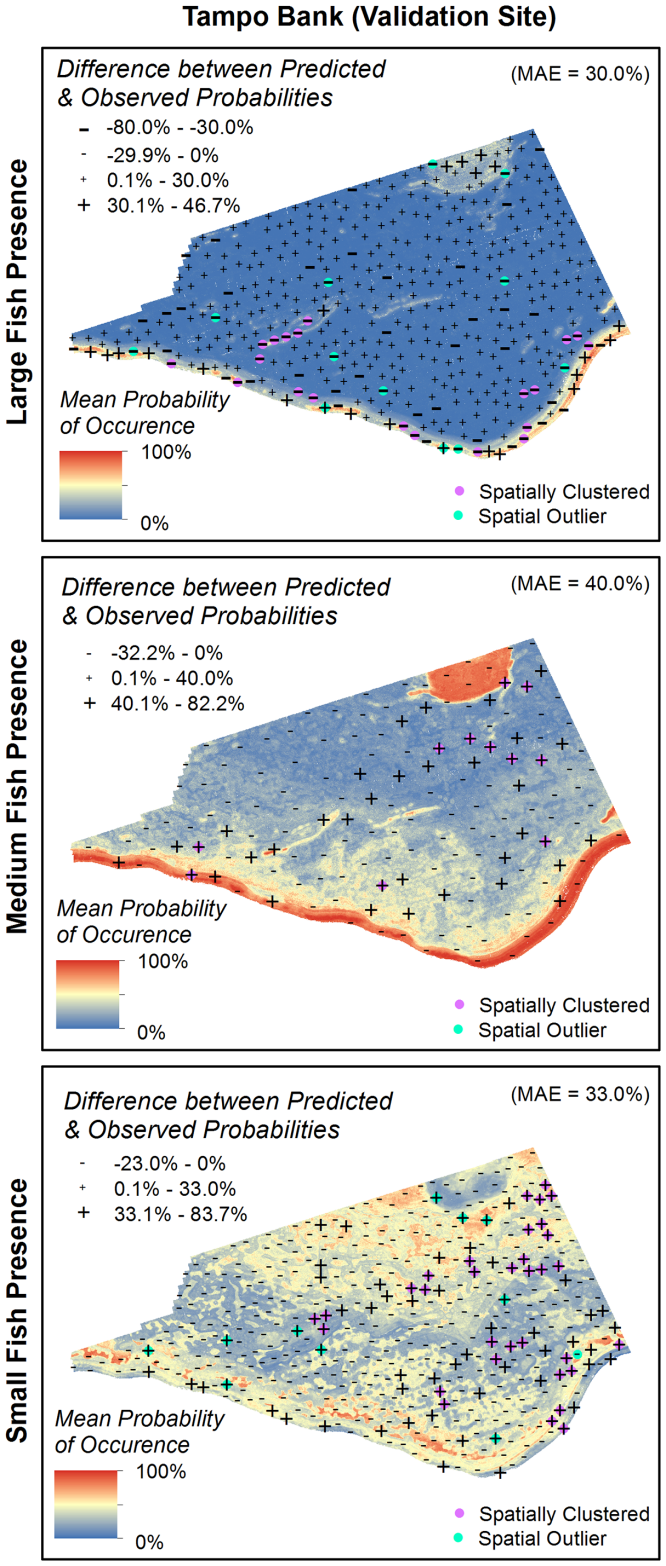
Errors for fish occurrence spatial predictions. The magnitude and spatial structure of the errors for the fish occurrence predictions were calculated by subtracting the predicted from observed probability of occurrence values. Negative values indicate that the model under-predicted, and positive values indicate the model over-predicted the probability of occurrence for large, medium and small fish. The error data was divided into classes based on the MAE. Spatial autocorrelation of the residuals were analyzed using Anselin Local Moran's I statistic. Analyzing the spatial location and arrangement of errors can be important because they may offer clues about missing ecological or biological variables.

Large fish were observed in 15% of the SBES records in the St. John Wedge area and 19% of the SBES records in the Tampo Bank area. In Tampo Bank, the AUC value for the large fish occurrence prediction (0.68±0.06) indicated ‘poor’ model performance (Fig. 5). The average difference between the predicted and observed probability of occurrence values was MAE = 30.0% and RMSE = 36.0% (Fig. 6).The majority (79.4%) of model errors were positive and ≤MAE, indicating that the BRT model more commonly over-predicted (vs. under-predicted) the probability of occurrence for large fish (Fig. 7). Negative errors (i.e., where the model under-predicted the probability of occurrence) were much less common (15.3%), but were always larger than the MAE. Large, positive errors comprised the remaining 5.3% of the model errors, and were located mainly along the shelf edge and over hard bottom in the northeast quadrant of Tampo Bank. Large negative errors were located throughout Tampo Bank, but were clustered primarily along the shelf edge and over a linear reef in the southwest quadrant of Tampo Bank. A description the partial dependence plots and influence of each predictor is not provided because the occurrence model for large fish performed poorly.

#### Medium and small fish

Medium and small fish were observed in 45% and 66% of the SBES records in the St. John Wedge area and 74% and 79% of the SBES records in the Tampo Bank area, respectively. In Tampo Bank, the AUC values for the medium fish (0.49±0.10) and small fish (0.55±0.07) occurrence predictions indicated that they performed no better than a random model (Fig. 5). This weaker model performance is also reflected in the larger MAE and RMSE values (Fig. 6), which were 3% to 15% higher for medium and small fish than for large fish (MAE = 40.0% and RMSE = 45.0% for medium fish, and MAE = 33.0% and RMSE = 41.0% for small fish). The majority of errors were negative and <MAE for medium and small fish (72.7% and 78.5%, respectively) (Fig. 7). All of the positive model errors were >MAE and clustered mainly on the insular shelf for both predictions. A description the partial dependence plots and influence of each predictor is not provided because the occurrence models for medium and small fish did not perform better than would be expected by random chance.

### Fish Density Models

#### Large fish

**Figure 8:**
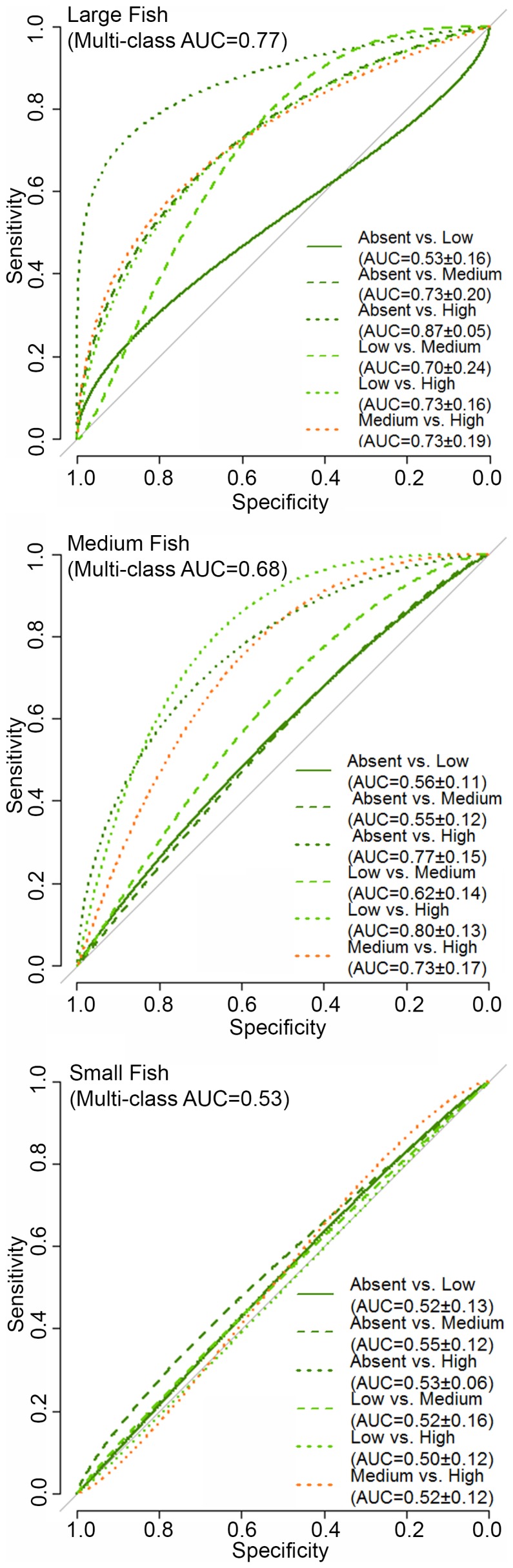
ROC curves for fish density predictions. These ROC curves were developed using the independent dataset in the Tampo Bank area. The multiclass AUC values are listed at the top of the figure for the large, medium and small fish predictions, and the AUC values (at the 95% confidence interval) are listed in the lower right hand corner for each pairwise comparison.

**Figure 9:**
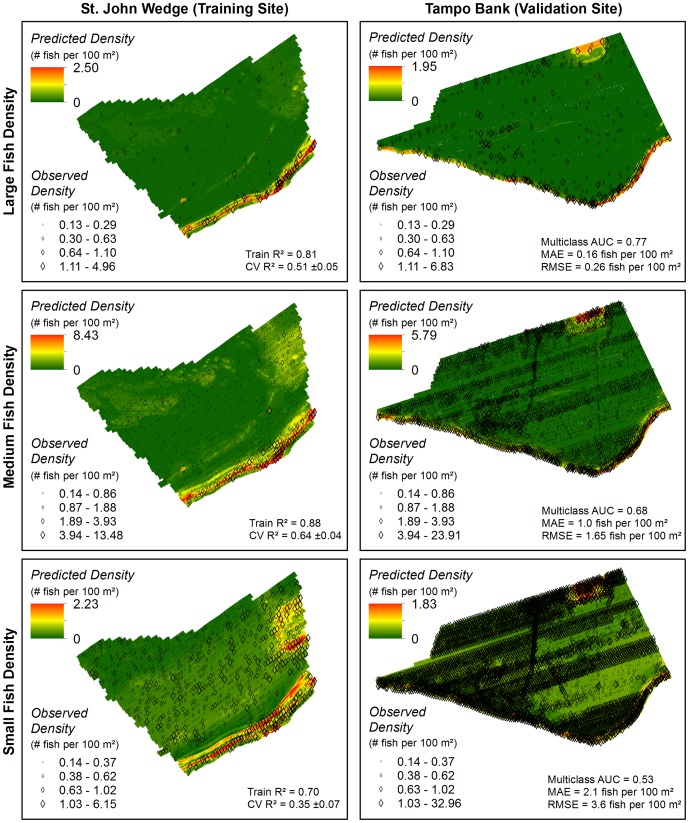
Fish density spatial predictions. Three spatial predictions denoting the density of large, medium and small fish were created for both the training and validation sites. The spatial outputs from 10 different BRT models were averaged to create the spatial predictions seen here. Metrics describing the performance and accuracy of these models and spatial predictions are located in the right left corner of each map.

**Figure 10:**
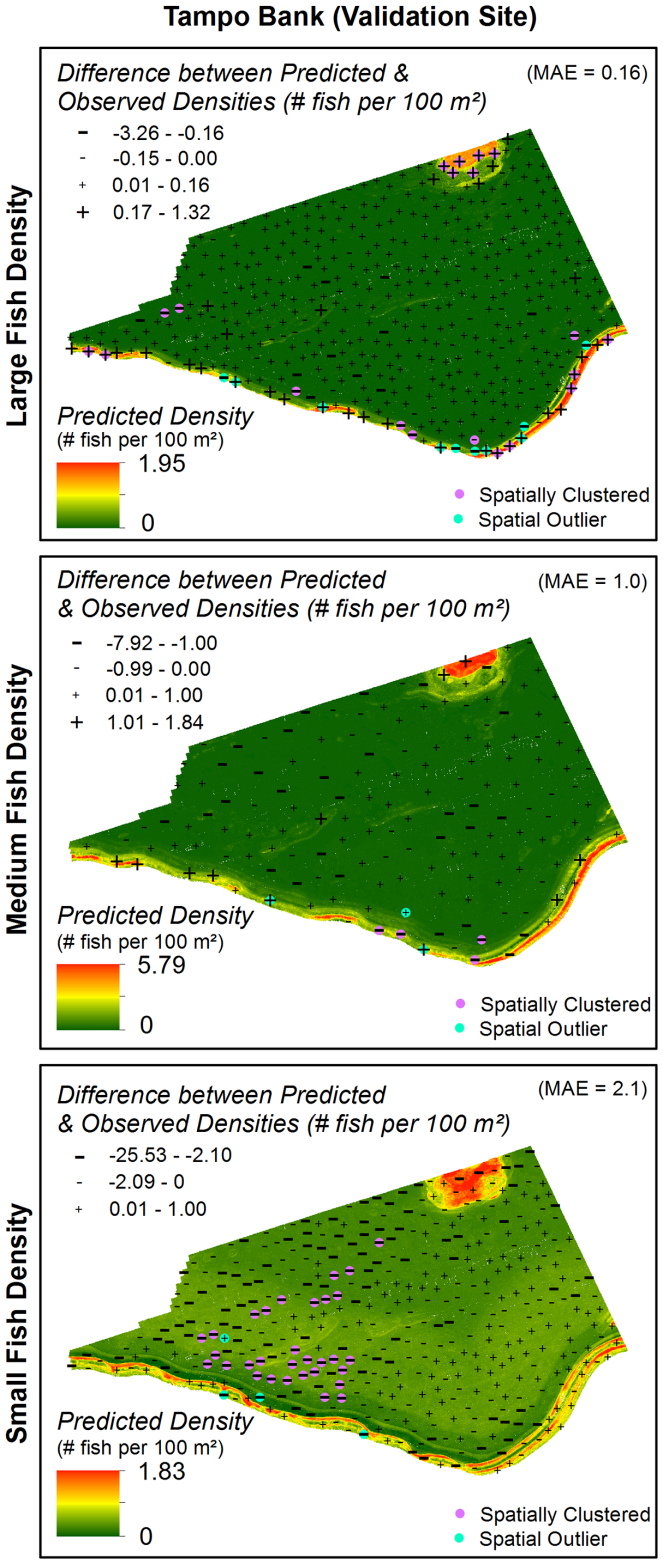
Errors for fish density spatial predictions. The magnitude and spatial structure of the errors for the fish density predictions were calculated by subtracting the predicted from the observed fish density values. Negative values indicate that the model under-predicted, and positive values indicate the model over-predicted the density of large, medium and small fish. The error data was divided into classes based on the MAE. Spatial autocorrelation of the residuals were analyzed using Anselin Local Moran's I statistic. Analyzing the spatial location and arrangement of errors can be important because they may offer clues about missing ecological or biological variables and their spatial structure.

High densities of large fish were rare (<1.8% of the SBES records) in both the St. John Wedge and Tampo Bank areas (Table 6). The multi-class AUC value (0.77) for the large fish density prediction indicated ‘good’ overall model performance (Fig. 8), outperforming the large fish occurrence model. Pairwise comparisons between density classes indicated that the BRT model was able to reliably distinguish the absent to low and the low density classes from the medium (AUC = 0.73±0.20; AUC = 0.70±0.24, respectively) and high (AUC = 0.87±0.05; AUC = 0.73±0.16, respectively) density classes. The model also reliably differentiated the medium from the high density class (AUC = 0.73±0.19), but not the absent to low from the low class (AUC = 0.53±0.16). The average difference between the predicted and observed large fish density values was MAE = 0.16 and RMSE = 0.26 fish per 100 m^2^ (Fig. 9).The majority (78.0%) of model errors were positive and <MAE, indicating that the BRT model more commonly over-predicted large fish densities (Fig. 10). Negative errors were much less common (12.7%), and were about equally above and below the MAE. Large, positive errors comprised the remaining 9.3% of the model errors, and were located mainly along the shelf edge and over hard bottom in the northeast quadrant of Tampo Bank. Large negative errors were located about equally on the insular shelf (n = 12) and along the insular shelf edge (n = 10) in Tampo Bank, although more clustering occurred along the shelf edge.

#### Medium fish

High densities were also uncommon for medium fish (i.e., <4.8%) in both project areas, although medium fish were more often found in low and medium densities (>17%) than large fish (Table 7). The medium fish density prediction performed poorly (multi-class AUC = 0.68; Fig. 8). Pairwise comparisons among the absent to low, the low and the medium densities classes also indicated that the medium fish density BRT model performed poorly or no better than a random model (AUC = 0.55 to 0.62). However, the BRT model was able to reliably distinguish the high density class from the absent to low, the low and the medium density classes (AUC = 0.77±0.15; AUC = 0.80±0.13; AUC = 0.73±0.17, respectively). The average difference between the predicted and observed medium fish density values was MAE = 1.0 and RMSE = 1.65 fish per 100 m^2^ (Fig. 9). Model errors were nearly equally split between being negative (43.5%) and positive (56.5%), indicating that the BRT model did not systematically under or over-predict medium fish densities (Fig. 10). The majority of errors (71.4%) were <MAE. Of the 28.6% of errors >MAE, more were negative (21.4%) than positive (7.1%). The large positive errors were located mainly along the shelf edge and in the northeast quadrant of Tampo Bank. The large negative errors were located in all four quadrants of the Tampo Bank area, but they were clustered along the shelf edge.

#### Small fish

Small fish were commonly found at medium and high densities (>19%) in both areas (Table 8). The BRT model was no better at predicting small fish densities than a random model (multi-class AUC = 0.53; Fig. 8), and could not reliably distinguish between any of the density classes (AUC<0.55). This weaker model performance is also reflected in the larger MAE and RMSE values (2.1 and 3.6 fish per 100 m^2^, respectively; Fig. 9). The majority (67.6%) of model errors were negative and clustered mainly in the southwest and northwest quadrants of Tampo Bank (Fig. 10). Approximately 31.6% of these negative errors were >MAE. Positive errors were all <MAE, and located mainly in the northeast and southeast quadrants. Given that the density models for small and medium fish both performed poorly, a description the partial dependence plots and influence of each predictor is not provided for either model.

**Figure 11:**
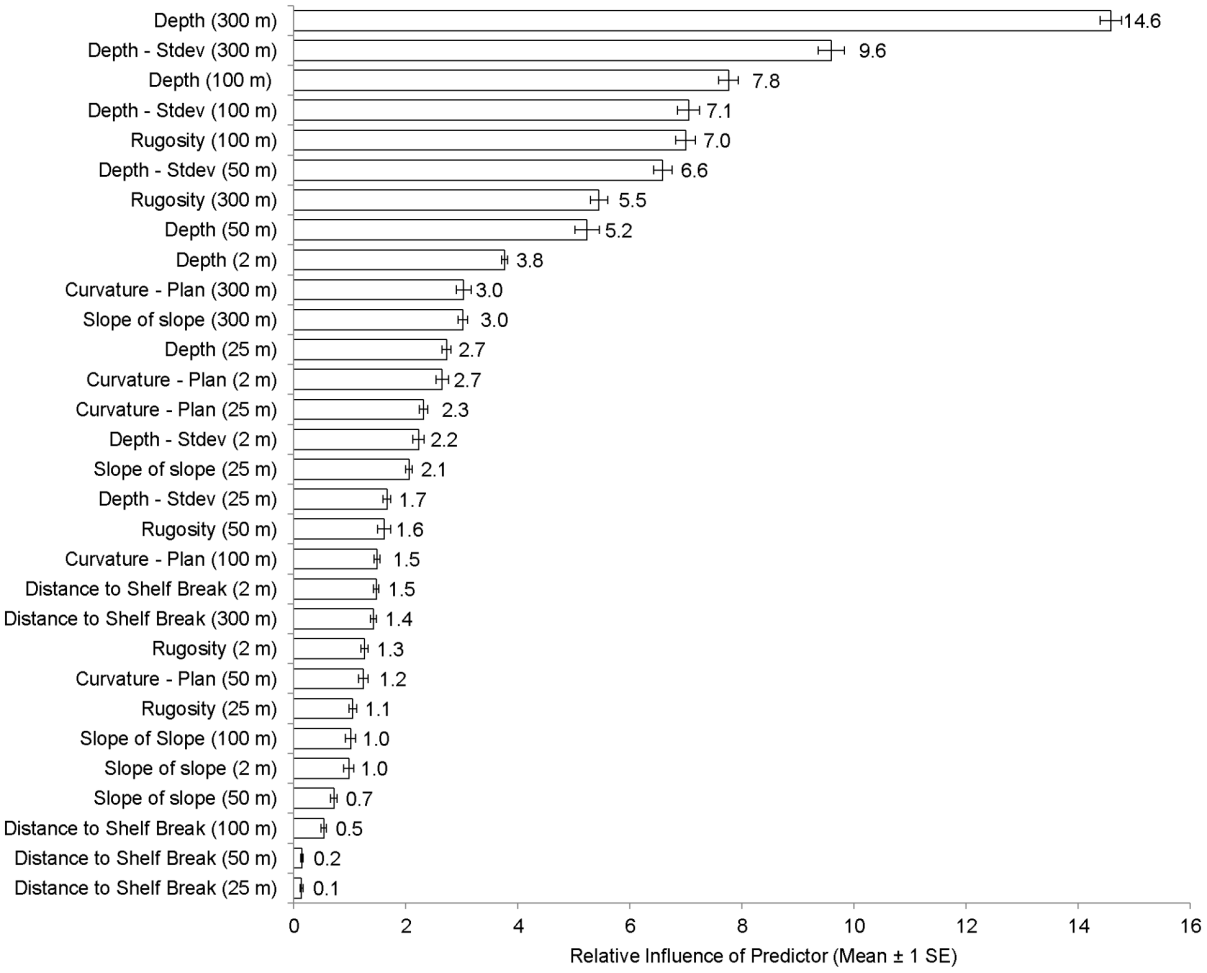
Influence of predictors on large fish density models. This figure shows the relative influence of each environmental variable on the large fish density spatial prediction. Cross validation data were used to calculate these values and standard errors, which were averaged 10 BRT model replicates.

**Figure 12:**
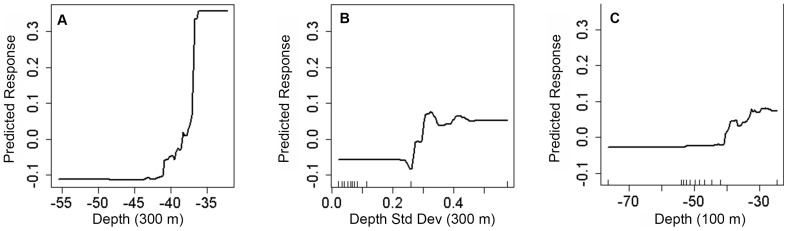
Partial dependence plots for large fish density models. Response curves for the three environmental variables that had the greatest influence on the prediction of large fish density. Collectively, these variables explain approximately 32% of the variance in the response data. All other variables were held at their average value.

High to medium densities of large fish were predicted along the shelf edge in both the St. John Wedge and Tampo Bank areas (Fig. 9). Low densities were predicted shoreward at the Tampo Bank area along hard bottom features with moderate amounts of structural complexity. Absent to low densities of large fish were predicted shoreward of the shelf edge over areas with low amounts of structure. Depth (at two different spatial scales) and standard deviation of depth were the top three most important environmental variables influencing the density and distribution of large fish (Fig. 11). These three predictors each explained between 7.8 and 14.6% of the variance in the large fish density data. All of these predictors had spatial scales ≥100 m, suggesting that the BRT density models were more heavily influenced by these variables at broad spatial scales. Partial dependence plots for these three predictors showed clear peaks and breakpoints in the response data (Fig. 12). When all other variables were held at their average values, large fish were more likely to occur at high densities where the seafloor was shallower (<38 m) and more complex (i.e., areas where the depth deviated by >0.29 m) (Figs. 12 a, b and c).

## Discussion & Conclusions

The novel integration of acoustic sensors offers a new approach to rapidly acquire spatial data across broad extents to identify both fish aggregations and areas of low fish occurrence. When combined with predictive modeling, it also offers a reliable method for predicting the density of large fish in areas where fish distributions have not been mapped. While fish acoustic systems are currently unable to identify fish species, they can collect many thousand observations (>5000 data points in each location) in a short amount of time (i.e., days to weeks versus months to years for SCUBA diver based surveys). These large acoustic datasets can support analysis—across a range of fish size classes and spatial scales—linking fish densities with seafloor structure and proximity to the insular shelf edge. They can also provide insights into geographic areas that are important for reef and non-reef associated species, helping coastal managers focus their efforts and limited resources on locations that may be of higher conservation value.

### Model Performance

#### Fish size classes

The model results indicate that we can reliably predict the density of large fish (in areas up to 100 m deep). Confusion between the absent to low and the low density classes was most likely due to the threshold chosen, and could be removed by merging the two classes. These results are comparable to those previously developed for shallow areas (<30 m) in a similar tropical coral reef ecosystem using fish distribution data from SCUBA diver surveys [14], [18], [33], [55], [56]. However, we were unable to produce an ‘acceptable’ model predicting the probability of occurrence for large fish. The large fish occurrence model may have performed better if we were able to divide the presence and absence data by species or trophic groups (instead of size class), as in previous studies.

Predictions for medium fish occurrence and for small fish occurrence and density were no more accurate than would be expected by random chance alone. Models for medium fish density performed somewhat better (particularly for the absent to low, the low and the medium versus high density classes), but their overall performance was still below the acceptable range. We attribute the poor performance of these models to the unequal prevalence of small to medium fish in the validation area versus the training area. No attempt was made to choose areas with similar prevalences because the main goal of this modeling effort was to predict fish distributions in un-surveyed (i.e., where animals' distributions and prevalences are unknown). However, this result highlights the need for caution when applying predictive models to new locations, and the need for an independent assessment of their accuracy before using them to make management decisions. In this case, it is difficult to know why smaller fish were more prevalent in the validation area. However, one possibility is the distinct shape of the shelf edge in the Tampo Bank area, which protrudes further out into deeper waters than the St. John Wedge area (Fig. 2). The importance of promontories (i.e., bends in the shelf edge, where the steep terrain protrudes into deeper waters) and shelf edge habitats are discussed in more detail in section 4.4.

In addition to unequal prevalences, we also attribute the poor performance of the medium and small fish models to the more even distribution and higher prevalence of smaller fish (45–79%) versus large fish (15–19%) overall. Smaller fish were most likely more prevalent because they experience less fishing pressure than larger fish, and they were more evenly distributed because they exploit a wider range of habitats than larger fish. The latter half of this explanation is supported by the fact that the most commonly seen small and medium fish groups included species with more varied habitat preferences and feeding habits than those found in the large fish class. Notably in Table 2, the most commonly seen large fish species were associated with only two types of habitats, whereas the most commonly seen medium and small fish species (Tables 3 and 4) were associated with three and four types of habitats, respectively.

#### Seafloor complexity predictors

The most influential factors for predicting large fish density were depth and variation in depth. Combined, these predictors explained 32% of the variance in the large fish density data. These influential predictors are similar to those identified by other reef fish modeling studies. Notably, depth explained over 10% of the variance in the occurrence of several fish species, and the abundance and biomass of piscivores in southwestern Puerto Rico [14], [18]. Slope of slope was also a common predictor for these individual species and community metrics [18], but it was not an influential factor in our models (<5% relative influence). Its influence may have been masked by the variation and overlap in habitat preferences among fish species grouped by size class. It is likely that both pelagic and demersal fish were included in our estimates of large fish occurrence and density, confounding the link between seafloor structure and large fish densities.

#### Spatial scale

The top model predictors for large fish density were important at relatively broad spatial scales (100 and 300 m). These relationships are similar to other modeling exercises, which also found linkages between larger fish and habitats at similarly broad spatial scales (i.e., 100, 200 m and 500 m) [14], [18], [34], [55]. However, some of these same studies also found that smaller fish responded to habitats at much finer (<25 m) spatial scales whereas we did not [18], [34]. One explanation for this difference may have to do with the timing of the fish surveys. Several of these studies used data collected during the day [18], [34], [55], whereas here, we used data collected between dusk and dawn. The time of day may be an important factor because many species make nocturnal migrations, feeding in habitats adjacent to structured reefs and hard bottom habitats [57], [58], [59]. Tagging studies have found that several reef fish species move 300 m or more diurnally [59] during these migrations.

#### Importance of the shelf-edge reefs

The relative influence of proximity to the shelf edge was relatively low compared to other factors (e.g., depth) for predicting large fish density. This reduced influence is most likely because depth and distance to shelf edge are highly correlated (i.e., the seafloor becomes deeper further from shore), and are most likely interchangeable as predictors. It remains that high fish occurrence and densities were observed and predicted at the shelf edge reefs in both St. John Wedge and Tampo Bank. High fish densities has also been noted at shelf-edge reefs in the Great Barrier Reef [60], [61] and other reef systems in the western Atlantic [62], [63], [64], [65]. The shelf-edge reefs are considered an important ecotone, where shelf waters containing land-based sources of nutrients converge with clear, oligotrophic ocean currents. Juveniles of many species, which use near-shore reefs and vegetated habitats, migrate to shelf edge reefs when they become adults presumably to rest, forage, and reproduce [66].

Our measure of distance from the shelf edge may also be a surrogate for other environmental or geophysical parameters that we did not measure. This explanation is supported by the fact that the biggest model errors (>MAE) for the large fish density prediction were spatially clustered along the shelf edge. This spatial clustering suggests that other physically and biologically important variables (e.g., nutrients, currents, thermoclines, prey abundances, fishing pressure) correlated with the shelf edge were missing from this modeling process. These variables were intentionally excluded from this study to investigate whether seafloor complexity would explain much of the variance in fish distributions. However, in future modeling iterations, additional oceanographic variables should be included at the very least, since nutrient supply and photic depth, combined with relatively stable, warm ocean waters appear to support high abundance of both oceanic and shelf species across a broad range of trophic guilds in this area [66]. Our observations of fish of all size classes show that densities are not the same along the entire shelf edge. Though we did not include a predictor to formally assess this pattern, higher densities were apparent along promontories in both regions. The promontories and submerged capes are notable geomorphologic features on the insular shelf of the U.S. Caribbean, and are common features where spawning aggregations for reef fish occur [6], [66]. These features may possess other qualities that support high densities of large fish during non-spawning periods. While we anticipated that shelf edge reefs and promontories would be important habitats, further research is needed to better understand the ecological processes behind these preferences.

### Management Implications and Future Developments

Splitbeam echosounders can rapidly survey fish distributions over large areas (10 s to 100 s km^2^) and at relatively fine spatial resolutions (<100 m^2^). This capability may make predictive models unnecessary in some cases. However in other cases, seafloor structure has been mapped in many areas without accompanying data describing fish distributions or densities. Model predictions could be used to provide first-order maps of large fish densities in these areas. These first order maps could potentially help managers focus their energies on areas that may be critical for large fish and that require additional study, as well as save resources by identifying broad areas that may not require visual surveys (e.g., over 93% of the sites presented here). These models could also be used to forecast how habitat use patterns for larger fish may change under different reef disturbance and flattening scenarios.

This study further emphasized reef complexity as an important geophysical feature in coral ecosystems, particularly at the ecotone of the insular shelf-edge. The shelf-edge habitats are also popular fishing grounds for pelagic and reef-associated species. Our findings at St. John Wedge and Tampo Bank have identified areas of high fish density that may benefit from long-term conservation and management actions to sustain fish populations. Visual surveys can also be conducted in these areas to better understand the environmental conditions attracting higher densities of fish, as well as to obtain better information on species comprising these assemblages.

This study also suggests that fish-seascape relationships and spatial predictions derived from fish acoustic surveys are similar to those derived from visual observations, although more research is needed directly comparing the two [14]. We are particularly encouraged by the performance of our model predicting large fish density. Coastal and fisheries managers are often most interested in the distribution of large, commercially valuable and vulnerable reef fish to identify essential fish habitat, including spawning aggregation sites. Identifying where large fish are most abundant will help coastal managers to prioritize sites and focus their efforts and limited resources on specific areas that may be of the highest conservation value. This type of targeted resource allocation will be particularly important as budgets are continually stretched and reef habitats become increasingly vulnerable, affecting the health and sustainability of reef fish populations. We propose wider use of these acoustic remote sensing tools, coupled with continued improvements in predictive modeling, to map and monitor fish aggregations in sensitive ecosystems.
